# Gene set analysis using sufficient dimension reduction

**DOI:** 10.1186/s12859-016-0928-6

**Published:** 2016-02-06

**Authors:** Huey-Miin Hsueh, Chen-An Tsai

**Affiliations:** Department of Statistics, National Chengchi UniversityZhinan Road, Taipei116, Taiwan, Taipei, 116 Taiwan; Department of Agronomy, National Taiwan University, No. 1, Section 4, Roosevelt Road, Taipei, 106 Taiwan

**Keywords:** Gene set analysis, Differential coexpression, Sufficient dimension reduction, Non-linear associations

## Abstract

**Background:**

Gene set analysis (GSA) aims to evaluate the association between the expression of biological pathways, or a priori defined gene sets, and a particular phenotype. Numerous GSA methods have been proposed to assess the enrichment of sets of genes. However, most methods are developed with respect to a specific alternative scenario, such as a differential mean pattern or a differential coexpression. Moreover, a very limited number of methods can handle either binary, categorical, or continuous phenotypes. In this paper, we develop two novel GSA tests, called SDRs, based on the sufficient dimension reduction technique, which aims to capture sufficient information about the relationship between genes and the phenotype. The advantages of our proposed methods are that they allow for categorical and continuous phenotypes, and they are also able to identify a variety of enriched gene sets.

**Results:**

Through simulation studies, we compared the type I error and power of SDRs with existing GSA methods for binary, triple, and continuous phenotypes. We found that SDR methods adequately control the type I error rate at the pre-specified nominal level, and they have a satisfactory power to detect gene sets with differential coexpression and to test non-linear associations between gene sets and a continuous phenotype. In addition, the SDR methods were compared with seven widely-used GSA methods using two real microarray datasets for illustration.

**Conclusions:**

We concluded that the SDR methods outperform the others because of their flexibility with regard to handling different kinds of phenotypes and their power to detect a wide range of alternative scenarios. Our real data analysis highlights the differences between GSA methods for detecting enriched gene sets.

**Electronic supplementary material:**

The online version of this article (doi:10.1186/s12859-016-0928-6) contains supplementary material, which is available to authorized users.

## Background

Gene set analysis (GSA) seeks to determine whether a predetermined gene set, in which the genes share a common biological function, is correlated with a phenotypic variable. In the past decade, many GSA methods have been proposed in scientific literatures. Goeman and Bühmann [1], Nam and Kim [2] Dinu et al. [3], and Maciejewski [4] have given thorough reviews and comparisons of previous GSA methods. Usually GSA methods are classified as either self-contained (Q2) or competitive (Q1) methods. Self-contained GSA methods have been used to reveal the association between gene sets and the phenotype of interest without taking other genes into consideration. In contrast, competitive GSA methods aim to provide the relative significance of a gene set when compared with available genes outside the gene set. Some methods use a parametric model to find the significance, while most methods use a resampling technique to obtain a nonparametric p-value. Usually the resampling is conducted with sample randomization to capture the variation between biological samples. However, to find the relative significance in a competitive GSA, some authors propose a resampling with gene randomization. Maciejewski [4] recently concluded that to have an organization similar to that of the actual biological study, the researchers should employ sample randomization. Here we aim to propose a self-contained method with sample randomization.

There are many ways to measure the association between a gene set and a phenotype. The attribute of the phenotype is a key point. When the phenotype is categorical, very often researchers focus on detecting differences among mean patterns of genes across distinct phenotypic groups. For example, with a binary phenotype, many methods make use of the conventional two-sample t-test, see Subramanian et al. [5], Tian et al. [6], Efron and Tibshirani [7], Irizarry et al. [8], Jiang and Gentleman [9] and so on. However, these approaches do not take the interaction between genes into consideration. To accommodate the correlations, Kong et al. [10] considered Hotelling’s test statistic of principle components, and Tsai and Chen [11], Chien et al. [12] suggested using the MANOVA approach. All these approaches test against the specific hypothesis that the gene set has common means across groups. They give satisfactory results when the gene set has a differentially expressed mean pattern. However, overemphasizing the first moments and ignoring other important information may result in a loss of power.

In addition to mean the second moments, including variance and correlation, have received more and more attention from researchers. A set of genes, being coexpressed across different biological samples, is said to be coexpressed. The network formed by coexpressed genes are of biological interest, since it provides evidence that these genes are functionally related, see Stuart et al. [13], Zhang and Horvath [14]. Furthermore, genes that have different coexpressions across groups are said to be differentially coexpressed. According to Cho et al. [15] differential coexpression analysis is helpful to explore key biological processes stimulated by changes in experimental conditions. Choi et al. [16] attempted to find the functional changes that accompany a comparison of two constructed coexpression networks under different biological conditions from ten published microarray data sets. Given a pre-determined gene set, Choi and Kendziorski [17] proposed a Gene Set Coexpression Analysis (GSCA) to identify differentially coexpressed gene sets. Rahmatallah et al. [18] developed the Gene Sets Net Correlations Analysis (GSNCA), which claims to account for the complete correlation structure of gene set analysis. The method for Evaluation of Dependency DifferentialitY (EDDY) proposed by Jung and Kim [19] also compares the joint probability distributions found in different conditions for a complete, thorough detection. Rahmatallah et al. [20] employed several minimum-spanning tree-based non-parametric multivariate tests to detect complex and specific alternative hypotheses.

Many microarray experiments involve more than two biological conditions, such as dose levels, time points, or treatment combinations; some even consider continuous phenotypes. To date, only a few of the previously developed GSA methods are able to handle either a categorical or a continuous phenotype. For example, the Gene Set Enrichment Analysis (GSEA) by Subramanian et al. [5], the methods by Tian et al. [6] and the global test (GT) by Goeman et al. [21]. Nevertheless, the other methods introduced in previous paragraphs are for the most best-suited for handling binary phenotypes. The linear combination test (LCT) by Dinu et al. [22] and its extended non-linear combination test (NLCT) by Wang et al. [23] are recently proposed GSAs specifically for continuous phenotypes. GSEA, LCT and NLCT assess the association between a gene set and a continuous phenotype using the Pearson correlation coefficient. Alternatively, GT is a score test for the random effect under a generalized linear model. On the other hand, when the phenotype is not binary, identifying coexpressed gene sets becomes more difficult due to limited observations in a genomic experiment. The previously mentioned GSCA method can deal with multiple phenotypic responses, while GSNCA is only suited to deal with a binary phenotype.

It can be seen that existing GSA methods are developed with respect to a particular alternative hypothesis, either of differential mean or of differential coexpression. To discover broader alternative spaces, this study aims to develop methods that can capture more information regarding the association between gene sets and phenotypes of interest. The proposed methods can be used as an initial screening in gene set analysis. When a significance appears, researchers can further investigate the source of deviation by using previously reviewed methods to determine whether there is a differential mean expression, a differential coexpression, or both. Further, our methods have wide applications in the sense of being suitable for binary, categorical or continuous phenotypes.

Sufficient dimension reduction (SDR) is an informative data reduction methodology used in regression analysis. Suppose *X* are *p*×1 predictors, *Y* is a univariate response, and the conditional distribution *Y*|*X* is the research of interest. Suppose there exists a *p*×*d* matrix *η*, where *d*≤*p*, such that *Y*|*X* and *Y*|*η*^*T*^*X* have the same probability distribution. Then the column space of *η* is called a dimension reduction subspace, which contains sufficient information of the association between *X* and *Y*, see Li [24]. The subspace always exists and is not unique. The so-called central subspace is the intersection of all dimension reduction subspaces, if the intersection is also a dimension reduction subspace. This subspace is the most compact and informative subspace. One major goal of SDR is to find the central subspace or its subspace.

Several authors proposed the use of different slicing and inverse regression analysis to find a subspace of the central subspace. The major difference is the kernel matrix used to estimate the central subspace. Table 1 in Bura and Yang [25] provides a thorough list of the SDR kernel matrices and the corresponding estimations of existing methods. Among them, the two most popular methods are the sliced inverse regression (SIR) by Li [24], and the sliced average variance estimation (SAVE) by Cook and Weisberg [26]. The kernel used in SIR is the covariance of the conditional mean of *X* given *Y*, which detects the deviation between the conditional mean and the marginal mean of *X*. On the other hand, the SAVE detects the deviation between the conditional covariance of *X* given *Y* and the marginal covariance of *X*. It has been shown in Cook and Lee [27] that the subspace found by SIR is contained in the subspace found by SAVE. More information about the association between *X* and *Y* is captured by applying SAVE. In this article, we employ the SAVE method for gene set analysis.

**Table 1:** Empirical type I error rates of eight GSA tests at *α*=0.05 for data of two biological conditions

Sample size		I. Homogeneity			II.Heterogeneity		
*n*	Methods	*p*=20	*p*=100	*p*=200	*p*=20	*p*=100	*p*=200
20	GSEA	0.112	0.108	0.096	0.142	0.096	0.102
	GT	0.048	0.058	0.048	0.046	0.044	0.042
	MVAT	0.058	0.074	0.048	0.038	0.052	0.048
	PCOT	0.050	0.046	0.044	0.038	0.052	0.028
	GSNCA	0.045	0.060	0.052	0.062	0.045	0.045
	GSCA	0.042	0.076	0.066	0.048	0.046	0.046
	SDR _*T*_	0.049	0.052	0.052	0.051	0.044	0.049
	SDR _*V*_	0.049	0.062	0.056	0.048	0.044	0.049
40	GSEA	0.094	0.086	0.092	0.138	0.114	0.092
	GT	0.054	0.044	0.044	0.058	0.050	0.042
	MVAT	0.042	0.036	0.048	0.062	0.038	0.056
	PCOT	0.056	0.058	0.058	0.046	0.064	0.056
	GSNCA	0.050	0.038	0.062	0.042	0.041	0.050
	GSCA	0.053	0.056	0.062	0.060	0.052	0.055
	SDR _*T*_	0.043	0.048	0.046	0.055	0.037	0.047
	SDR _*V*_	0.039	0.050	0.038	0.046	0.036	0.046
60	GSEA	0.138	0.112	0.090	0.136	0.114	0.098
	GT	0.046	0.058	0.052	0.052	0.068	0.044
	MVAT	0.052	0.062	0.050	0.042	0.048	0.038
	PCOT	0.058	0.074	0.048	0.046	0.056	0.060
	GSNCA	0.042	0.044	0.050	0.048	0.047	0.058
	GSCA	0.049	0.064	0.044	0.056	0.061	0.067
	SDR _*T*_	0.047	0.046	0.044	0.049	0.044	0.054
	SDR _*V*_	0.055	0.040	0.040	0.050	0.051	0.055

The determination of the dimension of the central subspace, the so-called structural dimension, is an important issue in SDR data analysis. Shao et al. [28] considered a point estimation of the dimension by sequentially applying the proposed marginal dimension test. Specifically, if the structural dimension is zero, there is no association between *X* and *Y*, which is the exact null hypothesis of GSA. In this article, this marginal dimension test for testing zero dimension is adopted to identify differentially expressed gene sets. A modified test that places more emphasis on means is also proposed. We conduct simulation studies for three scenarios of binary, three-class, and continuous phenotypes. Using simulated data sets, we study the performance of our proposed methods in terms of control of type I error and power in comparison with several existing methods. In addition, we also present the results of two real microarray datasets, the p53 dataset and GSE6956 dataset, for illustration. Significances of the deregulation of gene sets obtained from the Molecular Signature Database (MSigDB) of the GSEA website are measured using the proposed methods and the competing GSA methods.

The rest of the paper is organized as follows. In the Method section, the methodology of SAVE is briefly reviewed, and the marginal dimension test and its modification for GSA are then proposed. In the Results section, the proposed methods are evaluated and compared with other GSA methods using simulation studies and real microarray datasets. Lastly, discussion and a brief conclusion are provided at the end.

## Method

Suppose that *X* presents the gene expressions of a predetermined gene set of size *p*, and *Y* is the phenotypic response. In a self-contained GSA problem, we are interested in determining whether *X* is independent with *Y*. The following null hypothesis is tested:

$$H_{0}: X \text{is independent with}\,\, \textit{Y}. $$

When employing the slicing inverse regression analysis, *X* is standardized with respect to its marginal distribution, and denote *Z*=(*Z*_1_,…,*Z*_*p*_) as the standardized random vector. Assume a random sample {(*X*_(1)_,*Y*_(1)_),…,(*X*_(*n*)_,*Y*_(*n*)_)}, where (*X*_(*i*)_,*Y*_(*i*)_) are the original gene expressions and phenotype of the *i*-th subject respectively, *i*=1,…,*n*. Let $\bar {X}$ and $\hat {\Sigma }_{X}$ be the sample mean and the sample variance-covariance matrix of *X* respectively without taking *Y* into account. For *i*=1,…,*n*, let $Z_{(i)}=\hat {\Sigma }_{X}^{-1/2}\left (X_{(i)}-\bar {X}\right)$. Then *Z*_(1)_,…,*Z*_(*n*)_ are the *n* realizations of *Z*. It is known that the mean of *Z* is the zero vector and the covariance matrix of *Z* is the *p*×*p* identity matrix, *I*_*p*_. Next, the observations are classified into several disjoint groups, the so-called ’slices’, according to the value of *Y*. If *Y* is binary, multi-categorical, or discrete, there is a nature slicing. If *Y* is continuous, we consider a monotonic discretization. The subgroups (or slices) are formed by dividing the sample space of *Y*, a subset of *R*, into several disjoint intervals. Define the group/slice label variable as *S*. If there are *H* subgroups, *S*=*s*∈{1,2,…,*H*}. In the *s*-th slice, *S*=*s*, let $\hat {p}_{s}$ be the corresponding sample proportion, and let $\hat {\Sigma }_{Z|s}$ be the within-slice sample variance-covariance matrix of *Z*. In SAVE, the central subspace is the column space of the specific kernel matrix, *E*[*Var*(*Z*|*Y*)−*Var*(*Z*)]^2^, where *Var*(*Z*|*Y*) is the conditional covariance matrix of *Z* given *Y* in the inverse regression, and *Var*(*Z*) is the marginal covariance matrix of *Z*, which is equal to *I*_*p*_. The kernel matrix is estimated by $\sum _{s=1}^{H} \hat {p}_{s} \left (\hat {\Sigma }_{Z|s}-I_{p}\right)^{2}$.

The structural dimension, denoted by *d*, is defined as the dimension of the central subspace. If the gene set is not associated with the phenotype, the central subspace should be null and the structural dimension should be zero. Therefore, the problem is equivalent to testing the following hypothesis:

$$H_{0}: d=0 \,\,\text{versus}\,\,H_{1}: d>0. $$

Shao et al. [28] proposed the marginal dimension test with the following test statistic,

(1) $$ {\fontsize{9}{6} \begin{aligned} T=\sum\limits_{s=1}^{H} \hat{p}_{s} \; tr \left (\hat{\Sigma}_{Z|s}-I_{p} \right)^{2}= \sum\limits_{s=1}^{H} \hat{p}_{s} \left\{\sum\limits_{i=1}^{p} \sum\limits_{j=1}^{p} \left(\hat{\sigma}_{i,j|s}-{\sigma}_{i,j}\right)^{2} \right \}. \end{aligned}}  $$

In which, $\hat {\sigma }_{i,j|s}$ is the (*i,j*)-th element of $\hat {\Sigma }_{Z|s}$; and *σ*_*i,j*_ is the (*i,j*)-th element of *I*_*p*_. The null hypothesis is rejected if *T* is sufficiently large. Here we apply the marginal dimension test to assess the significance of the association between the gene set and the phenotype.

Explicitly, *T* assesses the weighted squared Euclidean distance between the within-slice sample covariance matrix and the pooled sample covariance matrix of *Z*. A significant difference results from the perturbation in the second moment of *Z* caused by the slicing based on the information of *Y*. In fact, the deviations in the first moment across slices also contributes to *T*. Denote the population version of *T* by *T*^′^, which is

$$T'=E\left[tr \left\{Var(Z|S) - Var(Z)\right\}^{2}\right]. $$

It can be shown that

$$\begin{array}{*{20}l}  T' =& \sum\limits_{i=1}^{p} E \left\{Var(Z_{i}|S)-E(Var(Z_{i}|S))\right\}^{2} + \\ &\sum\limits_{i=1}^{p} \sum\limits_{j \ne i}^{p} E\left\{Cov(Z_{i},Z_{j}|S)-E(Cov(Z_{i},Z_{j}|S))\right\}^{2}+\\ &\sum\limits_{i=1}^{p} \sum\limits_{j=1}^{p} \left\{E\left[(E(Z_{i}|S)-E(Z_{i}))(E(Z_{j}|S)-E(Z_{j}))\right]\right\}^{2}, \end{array} $$

where *Var*(*Z*_*i*_|*S*) is the conditional variance of *Z*_*i*_ given *S* and *Cov*(*Z*_*i*_,*Z*_*j*_|*S*) is the conditional covariance between *Z*_*i*_,*Z*_*j*_ given *S* for *i*≠*j*, and *i,j*=1,…,*p*. The first two terms show the deviations in the second moment. If the gene set has a constant mean across groups, the third term vanishes. However, when the conditional means of genes are independent of *S* but pairwisely uncorrelated, the third term is also negligible. It leads to a lack of power in detecting differential means. Hence, we proposed the following modified test statistic, which places more weight on the mean perturbation:

(2) $$\begin{array}{@{}rcl@{}} V = \sum\limits_{s=1}^{H} \hat{p}_{s} \; \left[ tr \left\{ \left(\hat{\Sigma}_{Z|s}^{1/2} - I_{p} \right)^{2} + \bar{Z}_{s} \bar{Z}_{s}^{T} \right\} \right]. \end{array} $$

In which, $\bar {Z}_{s}$ is the sample mean vector of *Z* in the *s*-th slice. The null hypothesis is rejected if a sufficiently large value of the test statistic is observed.

To evaluate the statistical significance, we perform a permutation test by using the proposed statistics. The phenotype labels of a given dataset are randomly permuted a thousand times and the SDR statistics are computed for each permuted dataset. An empirical distribution of each SDR statistic is then used to estimate a p-value with reference to the observed SDR statistic from the original data. At a significance level *α*, *H*_0_ is rejected if the *p*-value is not greater than *α*.

When a gene set is found to have a significant association with the phenotypic response, another question of interest is to find the hub genes in the set that contribute the most significance value. As per the definition in (1), *T* can be rearranged and expressed as a sum of *p* terms, $T=\sum _{i=1}^{p} T_{i}$, where

$$\begin{array}{@{}rcl@{}} T_{i}= \sum\limits_{s=1}^{H} \hat{p}_{s} \left[ \sum\limits_{j = 1}^{p} \left(\hat{\sigma}_{i,j|s}-{\sigma}_{i,j}\right)^{2} \right], \;\;i=1,\ldots, p. \end{array} $$

The statistic *T*_*i*_ sums up those deviations with regard to the *i*-th gene. As a result, marginal importance of the *i*-th gene can be evaluated on the value *T*_*i*_, or on the fraction *T*_*i*_/*T*. A gene plays an essential role if the value dominates that of most other genes in the set, or if the fraction exceeds some threshold. The significance of each individual gene can be also assessed using the previously mentioned permutation samples for significance by applying *T* in GSA. However, the significance is self-contained, not competitive, since it does not take other genes into consideration at the same time.

In this article, the gene set analysis is formulated as a specific problem in the sufficient dimension reduction analysis. Therefore, the proposed methods are referred to as the SDR methods. The proposed methods are applicable to single or multiple responses. In addition, they allow response variables to be binary, multi-class or continuous phenotypes. In the next section, we present a variety of simulation studies to compare the SDR methods with other existing methods, with regard to the performance of identification of differentially expressed gene sets.

## Results

### Simulation studies

In the following, the proposed methods are denoted by SDR _*T*_ and SDR _*V*_, corresponding to *T* in (1) and *V* in (2), respectively. The competing methods in the assessment include: (1) GSEA by Subramanian et al. [5] with R package *sigPathway*; (2) Global test (GT) by Goeman et al. with R package *globaltest*; (3) MVAT by Tsai and Chen [11]; (4) PCA-based test (PCOT) by Kong et al. [10] with R package *pcot2*; (5) GSNCA by Rahmatallah et al. [18]; (6) GSCA by Choi and Kendziorski [17]. The methods GSEA, GT, MVAT and PCOT are well-known GSA methods developed for differential expression, while GSNCA and GSCA are for differential coexpression. In the first and second simulations, differentially coexpressed gene sets with binary and three-class phenotype data are generated accordingly. Since PCOT and GSNCA are only applicable to comparisons of two data samples, these two methods are absent in the second simulation study. In the last scenario, where differentially expressed genes with a continuous phenotype are simulated, GSEA, GT, and our SDRs are compared with the LCT by Dinu et al. [22] under a linear model assumption, and NLCT by Wang et al. [23] under a non-linear model assumption. The p-values are based on 1,000 permutations. The simulation data are replicated 1,000 times in each model for the empirical type I error rate and empirical power in the null and alternative hypothesis, respectively.

### Binary phenotypic response

Our first simulation design adopts the setting used by Rahmatallah et al. [18] for two biological condition groups. In each replicate, we generate two gene expression matrices of equal sample size, *n*/2, from *p*-dimensional multivariate normal distributions (MVN) *N*(0,*Σ*_1_) and *N*(0,*Σ*_2_), respectively. Two different types of variance-covariance matrices are selected. The first homogeneous case assumes that all genes have an unit variance within each group. In contrast, in the heterogeneous case the variances of genes are randomly drawn from the uniform distribution *U*(1,5). With regard to the correlation structure, the genes in the first group are uncorrelated. Consequently, *Σ*_1_ is a *p*×*p* identity matrix in the homogeneous case and a diagonal matrix in the heterogeneous case. Under *H*_0_, the two covariance matrices are identical, i.e. *Σ*_2_=*Σ*_1_. In the alternative scenario, *Σ*_2_ is completely distinct from *Σ*_1_. In the diagonal, the variances of genes in the second group are also randomly generated from *U*(1,5), independent of the first group. In the off-diagonal, the first *γp* genes are equi-correlated with correlation *ρ* in the second group, where *γ*,*ρ*∈(0,1). In this simulation, the proportion of truly coexpressed genes, *γ*, is either 0.25, 0.5, 0.75, or 1; the inter-gene correlation *ρ* ranges from 0.1 to 0.9 with an increment of 0.1. Three gene set sizes are considered: relatively small (*p*=20), moderate (*p*=100), and relatively large (*p*=200). The total sample sizes *n* are 20, 40 and 60, respectively.

Table 1 shows the empirical type I error rates of the eight GSA methods at nominal level 0.05. Based on a simulation size of 1000, the standard error of the empirical type I error rate is.0069 when the true type I error rate is.05. Consequently, there is only 2.5 *%* of chance that the empirical error rate exceeds 0.064(=.05+(1.96)(.0069)) approximately. It can be seen that the empirical type I error rates of GSEA are all greater than 0.064. This method is too liberal. GSCA sometimes (4 times out of 18 scenarios) has an inflated type I error rate. In contrast, our two methods and GSNCA are good at controling the type I error rate in both homogeneous and heterogeneous cases. From this table, the heterogeneity in variations of genes does not affect the error rate of these methods.

**Fig. 1:**
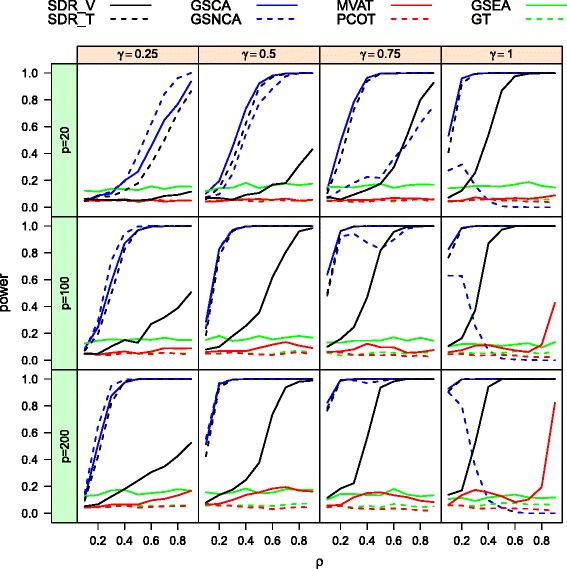
The power curves of MVAT, PCOT, GT, GSEA, SDRs, GSNCA, and GSCA in homogeneous cases for two biological conditions (*n*=40)

**Fig. 2:**
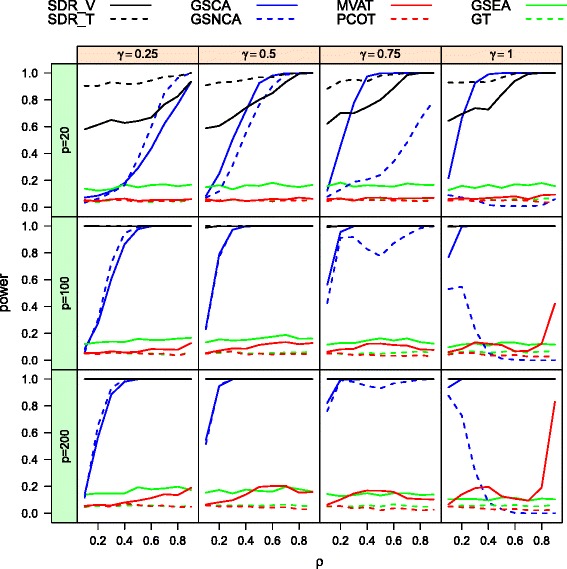
The power curves of MVAT, PCOT, GT, GSEA, SDRs, GSNCA, and GSCA in heterogeneous cases for two biological conditions (*n*=40)

The power curves, as functions of the inter-gene correlation *ρ*, of the eight methods for total sample size *n*=40 at nominal level 0.05 are provided respectively in Fig. 1 for the homogeneous case, and in Fig. 2 for the heterogeneous case. Note that the difference between two covariance matrices increases as *ρ* and *γ* increase. Hence, we expect to see a monotone trend in the power curves. Looking at Figs. 1 and 2, we observe that GSEA, GT, MVAT and PCOT, which were developed for detection of a mean difference, have unsatisfactory performance in terms of their ability to detect differential coexpression, as expected. Among these four methods, GSEA seems to be superior. However, it is important to note that its type I error rate is severely inflated in Table 1. In the following passage, we focus on the comparison of the four other methods: GSNCA, GSCA, SDR _*T*_, and SDR _*V*_. When *γ* is low, say *γ*=0.25, the GSNCA method outperforms the others in terms of statistical power. However, when the proportion *γ* is greater than 0.5, this method becomes less powerful than the other three methods, and its power curve is not monotone as the correlation deviates from zero. When all genes are pairwisely correlated in the second group, i.e. *γ*=1, the power decreases with inter-gene correlation and becomes powerless for large *ρ*. On the other hand, SDR _*T*_, SDR _*V*_, and GSCA have the expected trends in power, increasing with *γ* and *ρ*. SDR _*T*_ and GSCA are comparable and dominate SDR _*V*_ across different combinations of *γ* and *ρ*. The test SDR _*V*_ places more emphasis on mean difference and as a result suffers a power loss in detecting differential coexpression.

In the heterogeneous case, Fig. 2 shows that the power of SDR _*T*_ is much higher than the power of GSNCA and GSCA because it successfully detects the deviation in variances. SDR _*V*_ has comparable performance with SDR _*T*_ when the gene set size *p* is moderate to large. Again when the proportion of truly coexpressed genes is large (*γ*=0.75,1), the power of GSNCA does not increase with the inter-gene correlation *ρ*. As a result, SDR _*T*_, SDR _*V*_, GSCA, and GSNCA all demonstrate that they are good at identifying differential correlation of genes within a gene set. When a great proportion of genes are correlated, GSNCA should be applied with caution. In actuality, genes are likely to have differential variations in real gene expression data. Both of the proposed SDR methods have an advantage when dealing with differential variations of genes.

### Three-class phenotypic response

For each replicate, we generate three independent random samples of *p* gene expressions with equal sample size, *n*/3, from *p*-dimensional multivariate normal distributions (MVN) *N*(0,*Σ*_1_), *N*(0,*Σ*_2_), and *N*(0,*Σ*_3_), respectively. This simulates an experiment with three biological conditions. All the diagonal elements of the three covariance matrices are randomly generated from *U*(1,5). Furthermore, *Σ*_1_ is a diagonal matrix. Both *Σ*_2_ and *Σ*_3_ have the following form of a block diagonal matrix of equal size *p*/4:

$$\Sigma_{i} = \left[ \begin{array}{cccc} V_{1} & 0 & 0 & 0\\ 0 & V_{2} & 0 & 0\\ 0 & 0 & V_{3} & 0\\ 0 & 0 & 0 & V_{4} \end{array} \right], \,\,i=2,3. $$

Next, a mixed correlation structure between genes is adopted in each block. Within each block, 100*γ* percent of genes are equi-correlated with correlation *ρ*; otherwise, the genes are uncorrelated. In order to simulate differentially coexpressed genes, correlated genes inside each block are assigned to different positions for *Σ*_2_ and *Σ*_3_. Specifically, in every block the first *γp*/4 genes are correlated in *Σ*_2_, while the last *γp*/4 genes are correlated in *Σ*_3_.

**Fig. 3:**
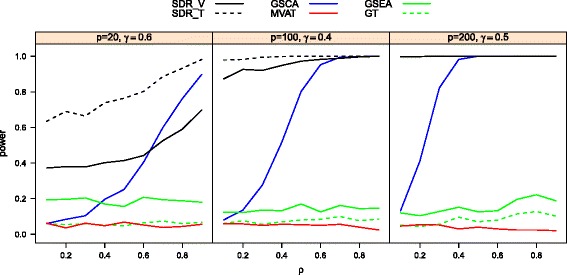
The power curves of SDRs, GSCA, MVAT, GT, GSEA in heterogeneous cases for three biological conditions (*n*=30)

Figure 3 provides the power curves of GSEA, GT, MVAT, GSCA, SDR _*T*_ and SDR _*V*_ for experiments with total sample size *n*=30 at selected combinations of *p*,*γ*. As in previous power studies, GSEA, GT, and MVAT lack the power to detect differentially coexpressed gene sets. The power of SDR _*V*_ is relatively low for small *p*, but it improves when the gene set size *p* increases. SDR _*T*_ outperforms other methods, even when the inter-gene correlation is small.

### Continuous phenotypic response

In this study, gene expressions are generated according to the following model: For *i*=1,…,*n*,

$$X_{i} \stackrel{i.i.d.}{\sim} \;MVN(0, \Sigma_{X}), $$

where the elements of the covariance matrix *Σ*_*X*_=(*ρ*_*i,j*_)_*p*×*p*_ are given by

$${\fontsize{8}{6}\begin{aligned} \rho_{i,j} = \left\{ \begin{array}{ll} 1, & 1 \le i=j \le p,\\ \rho, & 1 \le i \ne j \le p_{1},\\ \rho^{|i-j|}, & p_{1}+1 \le i \ne j \le 2p_{1}, \\ 0, & \text{otherwise.} \end{array}\right. \end{aligned}} $$

That is, all *p* genes have unit variance, and the first 2*p*_1_ of them are pairwisely correlated. The first *p*_1_ genes are equi-correlated pairwisely with correlation *ρ*. The correlation of the next *p*_1_ genes decreases as the distance between the two genes increases. Specifically, *ρ*=0,0.3,0.6,0.9 are selected.

For the null scenario, the continuous phenotype *Y*, being independent of *X*, is randomly drawn from *N*(0,1). We consider two alternative scenarios. The first is a traditional normal linear regression model : For *i*=1,…,*n*, given *x*_*i*_,

$$Y_{i}|x_{i} \; \sim \;\; N\left({x_{i}^{T}}\beta, 1\right). $$

The second alternative model is a non-linear model: For *i*=1,…,*n*, given *x*_*i*_,

$$Y_{i}|x_{i} \,\sim \,\, N\left(exp\left({x_{i}^{T}}\beta\right), 1\right). $$

In which, the regression coefficient vector is *β*=(*β*_1_,…,*β*_*p*_)^*T*^. Suppose that in both models the phenotype *Y* depends on ten genes, five belong to the first group of *p*_1_ genes, the other five belong to the next group of *p*_1_ genes. We randomly select 5 of the first group of *p*_1_ genes, and then produce their corresponding *β*_*j*_’s from *N*(*ν*,|*ν*|). Next, another 5 genes from the second group of *p*_1_ genes are randomly selected, and their corresponding $\phantom {\dot {i}\!}\beta _{j'}$’s are generated from *N*(−*ν*,|*ν*|). Aside from the ten selected genes, all other genes have zero regression coefficients. Several *ν*’s ranging from 0 to 2 are considered. We consider two equal slices for the SDR methods, i.e. $H=2, \hat {p}_{1}=\hat {p}_{2}=0.5$.

Table 2 reports the empirical type I error rates of GSEA, GT, SDR _*T*_, SDR _*V*_ and LCT at significance level *α*=0.01,0.05 for (*n,p*,*p*_1_)=(20,20,5), (30,100,20),(50,200,40). Based on a simulation size of 1000, the 97.5 *%* limit of the empirical type I error rate is.016 and.064 respectively, which corresponds to true error rate.01 and.05. Again GSEA is found to be too liberal in terms of a poor control of type I error rate. In contrast, GT, LCT, SDR _*V*_, SDR _*T*_ preserve type I error rates, while SDR _*T*_ can have a slightly inflated type I error rate for independent cases.

**Table 2:** Empirical type I error rate of five GSA tests at *α*=0.05 for data with a continuous phenotype

		*α*=0.01				*α*=0.05			
(*n,p*,*p*_1_)	*ρ*	0.0	0.3	0.6	0.9	0.0	0.3	0.6	0.9
(20,20,5)	GSEA	0.030	0.040	0.033	0.053	0.126	0.154	0.164	0.187
	GT	0.011	0.008	0.010	0.009	0.049	0.039	0.043	0.053
	LCT	0.010	0.008	0.014	0.008	0.047	0.047	0.066	0.037
	SDR _*V*_	0.009	0.008	0.013	0.017	0.048	0.044	0.052	0.046
	SDR _*T*_	0.014	0.011	0.010	0.017	0.055	0.053	0.049	0.055
(30,100,20)	GSEA	0.021	0.036	0.035	0.057	0.110	0.147	0.178	0.188
	GT	0.010	0.013	0.005	0.009	0.053	0.044	0.050	0.067
	LCT	0.013	0.010	0.008	0.017	0.060	0.046	0.047	0.052
	SDR _*V*_	0.009	0.015	0.010	0.012	0.043	0.047	0.045	0.042
	SDR _*T*_	0.008	0.014	0.016	0.009	0.048	0.052	0.051	0.042
(50,200,40)	GSEA	0.018	0.047	0.050	0.058	0.096	0.159	0.184	0.197
	GT	0.004	0.015	0.007	0.008	0.038	0.050	0.061	0.054
	LCT	0.012	0.012	0.014	0.013	0.056	0.052	0.060	0.042
	SDR _*V*_	0.010	0.008	0.007	0.008	0.059	0.039	0.045	0.048
	SDR _*T*_	0.018	0.008	0.009	0.008	0.072	0.050	0.044	0.050

**Fig. 4:**
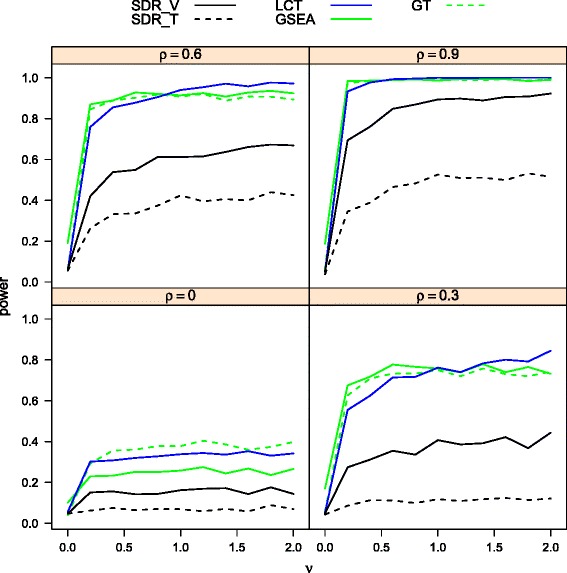
Power comparison (*n*=20, *p*=100, and *p*_1_=20) of SDRs, LCT, GT, GSEA for linear relationship between phenotype and gene set

**Fig. 5:**
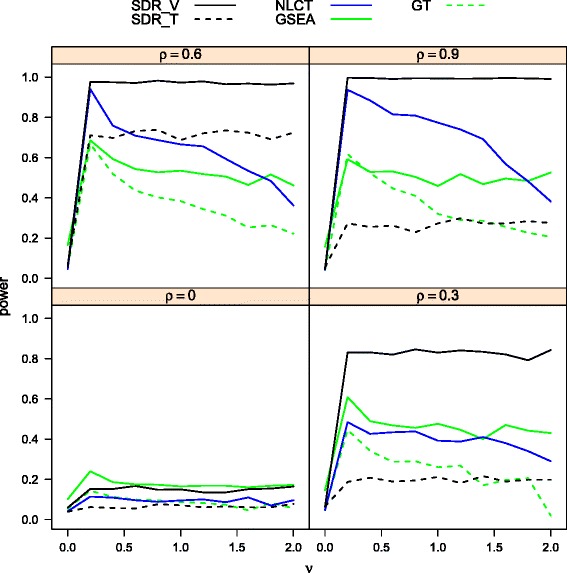
Power comparison (*n*=20, *p*=100, and *p*_1_=20) of SDRs, NLCT, GT, GSEA for nonlinear relationship between phenotype and gene set

Figures 4 and 5 illustrate the power curves of the methods being investigated under linear and non-linear models, respectively, for *n*=20,*p*=100,*p*_1_=20, and *α*=0.05. Since LCT was developed under a linear model assumption, it is not suitable for comparisons under non-linear models. Hence, in the non-linear scenario, we consider NLCT, which is a non-linear version of an extended LCT, as an alternative to LCT in the comparison. Figure 4 shows that SDR _*T*_ and SDR _*V*_ are dominated by GSEA, GT and LCT in the linear model. The three dominating methods evaluate the significance of a gene set by its linear correlation with the phenotype. Hence they demonstrate excellent performance in a linear model, which has a strong link to a high linear correlation. The proposed SDR methods focus on the information of the conditional distribution of phenotype given a set of genes. The association under investigation is not limited to the linear correlation. However, as stated previously, accounting for a broader class of alternatives results in a loss of power with respect to local alternatives. Among the two SDRs, SDR _*V*_ performs better, because its extra attention on the mean increases the power to detect a deviation in the pattern.

From Fig. 5, it can be seen that SDR _*V*_ has substantially higher power than other methods in the non-linear model with NCLT coming in second. SDR _*T*_ and GT are dominated by SDR _*V*_ and NCLT. SDR _*T*_ has acceptable performance only at *ρ*=.6. GSEA still suffers from a poor control of type I error rate in the continuous case.

### Analysis of the p53 dataset

Next we investigate the performance of the GSA methods with respect to the p53 microarray dataset. The p53 cancer data set is frequently used for GSA illustrations (e.g. [5, 29]) and publicly available at the GSEA website (http://www.broad.mit.edu/gsea/datasets.jsp). The p53 dataset seeks to identify targets of the transcription factor p53 from 10,100 gene expression profiles in the NCI-60 collection of cancer cell lines. The mutation status of the p53 gene has been reported for 50 of the NCI-60 cell lines with 17 normal and 33 mutation samples. The p53 protein is a transcription factor that plays a major role in suppressing cancer. We perform GSA comparisons on the C2 curated gene sets in the Molecular Signatures Database (MSigDB) on the GSEA website. The MSigDB contains over 6000 gene sets of a variety of functional types. We first discard genes in C2 pathways which do not exist in the p53 dataset and only keep gene sets of sizes between 10 and 500, resulting in 2533 gene sets to be considered in this study.

**Fig. 6:**
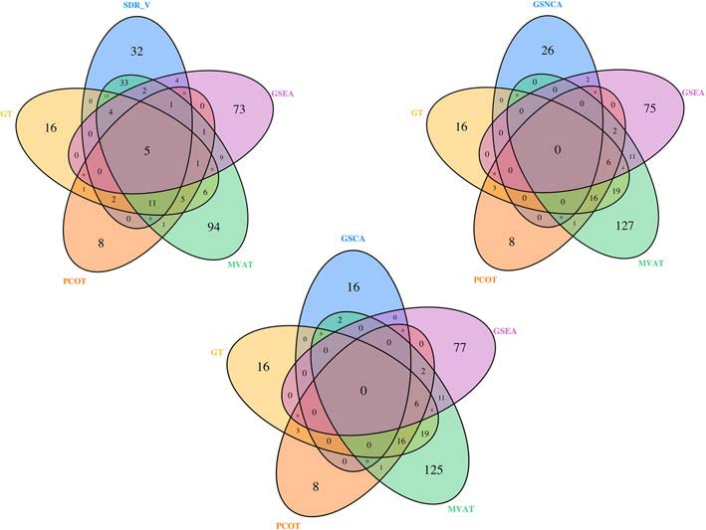
Venn diagrams of significant gene sets for each of the three GSA methods, SDR _*V*_, GSNCA, GSCA, and the other four GSA methods using the P53 cancer dataset at the 0.01 significance level

We compare the p-values obtained via th eight methods. Table 3 shows the number of differentially expressed gene sets identified at varying significance levels. Looking at the table, MVAT, SDR _*V*_, GSEA find most significant pathways while GSNCA and GSCA find the least. Among the two proposed tests, using SDR _*V*_ leads to more discoveries than using SDR _*T*_. These findings imply that more gene sets express differentially in the mean, rather than in the correlation structure, across the two distinct p53-mutation status groups. The Venn diagrams in Fig. 6 show the common pathways detected by each of SDR _*V*_, GSNCA, GSCA, and the other four methods: GSEA, GT, MVAT and PCOT, at significance level *α*=0.01. It shows that SDR _*V*_ and the other four methods find more significant gene sets in common. However, the findings of GSNCA and GSCA rarely overlap with the findings of the other four methods. Using one of the methods alone may miss the deviation from other angles in gene expressions.

**Table 3:** Number of differentially expressed gene sets identified by eight GSA methods for the p53 dataset

*P* −value	SDR _*T*_	SDR _*V*_	GSEA	GT	MVAT	PCOT	GSNCA	GSCA
≤0.001	10	40	12	15	44	8	5	2
≤0.01	45	107	100	64	186	36	28	18
≤0.05	199	329	413	226	627	143	159	100

**Fig. 7:**
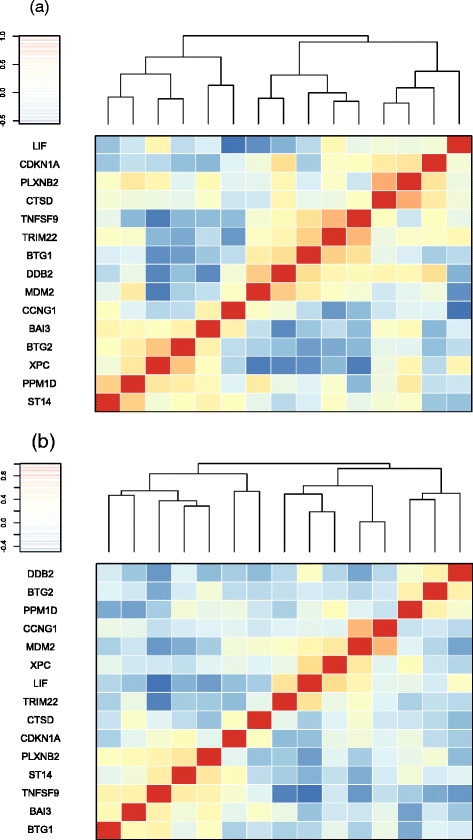
Image plots of correlation matrices for gene set “AMUNDSON DNA DAMAGE RESPONSE TP53” in p53 dataset. The Pearson correlation coefficients among **a** normal and **b** mutation samples are shown in an image plot with a hierarchical clustering dendrogram

Among the C2 curated gene sets, we highlight a particular gene set associated with DNA damage, AMUNDSON_DNA_DAMAGE_RESPONSE_TP53. This gene set is involved in the apoptosis and DNA damage response to a robust p53-dependent pattern of induction. Interestingly, the gene set was identified as a highly differentially expressed gene set by SDR _*V*_ with *p*-value < 0.001, but was not identified as significant by either GSCA (*p*-value = 0.60) or GSNCA (*p*-value = 0.58). To focus on the 15 genes in this gene set, a Pearson correlation matrix is used to investigate the dependence structure between genes for normal and mutation groups. Figure 7 displays the image plot of the reordered correlation matrix using hierarchical clustering to visualize the degree of association between genes. According to the plot, there is a clear difference in the correlation structure between two conditions. This indicates that the SDR _*V*_ method is able to identify more enriched gene sets with differential coexpression for further investigation.

### Analysis of the GSE6956 dataset

In the second real example, the gene expression profiles of primary prostate tumors from 33 African-American patients using the Affymetrix microarray platform are analyzed, see Wallace et al. [30]. Each profile contains the expression levels of 12,500 genes. We downloaded the gene expression data from the NCBI GEO database (Edgar et al. [31]) with accession ID GSE6956. Recently, a thorough review on relevant literatures published from 1991 to 2012 on PubMed by Allott, Masko and Freedland [32] concludes the existence of a link between obesity and aggressive prostate cancer. It is known that Leptin, a hormone produced by adipose cells, plays an important role in regulating appetite and body weight. In an earlier article, Freedland and Aronson [33] mentioned that leptin is a potential prognostic marker for prostate cancer patients because they found that increased leptin levels in plasma or serum are associated with the development of prostate cancer. Specifically, the expression level of the human leptin gene (LEP) was used as a continuous-type phenotype, see Dinu et al. [22]. The goal of this analysis is to identify pathways that are significantly associated with LEP for prostate cancer patients. We perform GSA comparisons on the C2 curated gene sets in the Molecular Signatures Database (MSigDB) on the GSEA website. The MSigDB contains over 6000 gene sets of a variety of functional types. We first discard genes in C2 pathways which do not exist in the dataset and only keep gene sets of sizes between 10 and 500, resulting in 2,595 gene sets to be considered in this study. The proposed SDRs methods consider two equal slices.

**Fig. 8:**
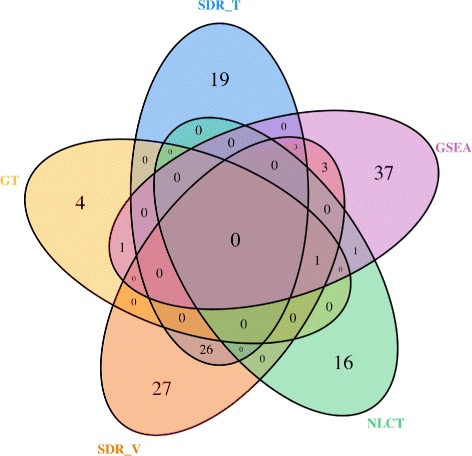
Venn diagrams of significant gene sets for five GSA methods using the GSE6956 dataset at the 0.01 significance level

Table 4 shows the number of differentially expressed gene sets identified by each method at significance levels 0.01, 0.05, and 0.10. Looking at the table, SDR _*V*_, SDR _*T*_, and GSEA find more significant pathways. Although NLCT claims that it is capable of detecting non-linear associations, it identifies fewer significant pathways in this example. The Venn diagrams in Fig. 8 show the common pathways detected by the five methods at significance level *α*=0.01. Except for SDR _*T*_ and SDR _*V*_, which identify over 50 *%* common significant gene sets, there are few overlapping gene sets of pairwise GSA methods.

**Table 4:** Number of differentially expressed gene sets identified by five GSA methods for the GSE6956 dataset

*P* −value	SDR _*T*_	SDR _*V*_	GSEA	GT	NLCT
≤0.01	48	60	46	6	18
≤0.05	211	249	235	45	104
≤0.10	419	494	455	94	259

## Discussion

Since Subramanian et al. [5] proposed the concept of gene set enrichment analysis (GSEA), many self-contained GSA have been proposed to identify enriched gene sets or pathways. Most previous studies focus on testing the enrichment of gene sets with a differential mean expression or differential coexpression. In this paper, we propose two self-contained tests for gene set analysis by adopting the sufficient dimension reduction paradigm. The information that the proposed SDR tests acquire include the deviations in mean, variation and correlation structure. As a consequence, these methods are more flexible in terms of being able to detect a wide variety of alternative scenarios.

Through numerical studies, we compare the suitability of proposed SDR methods with that of other existing GSA methods to test differential expression with a continuous phenotype and also to test differential coexpression with a categorical phenotype. Overall the SDR methods yield satisfactory performance. More specifically, SDR _*T*_ excels at detecting differential variation and/or coexpression while SDR _*V*_ is recommended for differentially expressed gene sets. However, as a trade-off, their statistical powers may be dominated locally by other methods developed under specific alternatives. Another shortcoming is the increased computational burden, because the tests involve calculating the group-wise or slice-wise covariance matrices.

In most gene expression data sets, the number of subjects *n* is much fewer than the number of genes *p*. It leads to a singular sample covariance matrix of *X*. Consequently, data standardization becomes difficult. One solution is to apply another covariance matrix estimation, which is guaranteed to always be non-singular. For example, the shrinkage covariance matrix proposed by Sch$\ddot {a}$fer and Strimmer [34]. Alternatively, since the aim here is to determine the structural dimension, one can simply skip the standardization step. Consider the following modified test statistics,

$${\fontsize{9}{8}\begin{aligned} T^{*}&=\sum\limits_{s=1}^{H} \hat{p}_{s} \; tr \left(\hat{\Sigma}_{X|s}-\hat{\Sigma}_{X} \right)^{2}, \\ V^{*} &= \sum\limits_{s=1}^{H} \hat{p}_{s} \; \left[ tr \left\{ \left(\hat{\Sigma}_{X|s}^{1/2} - \hat{\Sigma}_{X}^{1/2}\right)^{2} + \left(\bar{X}_{s}-\bar{X}\right) \left(\bar{X}_{s}-\bar{X}\right)^{T} \right\} \right], \end{aligned}} $$

where $\hat {\Sigma }_{X|s}=(\hat {\sigma }_{i,j|s})$ is the sample covariance matrix of *X* in the *s*-th slice, *s*=1.…,*H*; and $\hat {\Sigma }_{X} = (\hat {\sigma }_{i,j})$ is the the sample covariance matrix of *X* calculated from the pooled sample.

The proposed methods are applicable to single, multiple, categorical, and continuous phenotypes. With a continuous response, the slicing/discretization is employed to reduce the sparsity, and this may result in a loss of statistical power. Li [24] indicated that the slice number may affect the asymptotic property of the estimate, although in their simulation study the effect is not significant. Becker and Gather [35] showed that different slice numbers produce different estimates for the structural dimension. They recommend a reasonable slice number, about 0.1*n*. We have conducted a simulation study to investigate the effect of slice numbers. Simulation setting and results are provided in detail in the Additional file 1. We find that SDR _*V*_ is robust with respect to the slice number, while SDR _*T*_ is not. When employing SDR _*T*_, researchers are advised to use various slice numbers. With limited samples, as is the case in a real genomic study, using fewer slice number yields better performance.

In the real examples, different methods very often find different significant gene sets. Similar findings can be seen in Wu and Lin [36]. This reflects the fact that each method is constructed under different alternative hypothesis and uses different approaches to search for significant gene sets. Even though sufficient dimension reduction analysis aims to gain the most thorough information about a regression model. However, the space estimated by developed techniques, such as SIR and SAVE, is shown only as a subspace of the central subspace. This indicates that some informative part of the central subspace may be still missing, and it also explains why the proposed methods are not able to provide an exhaustive list of significant pathways in the examples.

## Conclusions

We have introduced two new GSA methods based on the concept of sufficient dimension reduction, which has the ability to capture sufficient and essential structural information in gene sets. The proposed SDR methods provide increased statistical power and can accommodate both categorical and continuous phenotypes in order to assess the significance of a given gene set.

## Additional file

Additional file 1
**The effect of slice numbers on SDR method.** (PDF 126 kb)
