# Supplementary material for: Gene set analysis using sufficient dimension reduction
Source: BMC Bioinformatics. 2016 Feb 6;17:74. doi: 10.1186/s12859-016-0928-6 (PMC4744442; doi:10.1186/s12859-016-0928-6)

Supplementary of "Gene Set Analysis Using Sufficient Dimension Reduction"

Huey-Miin Hsueh[1] and Chen-An Tsai*[2]

Email: Huey-Miin Hsueh - hsueh@nccu.edu.tw; Chen-An Tsai - catsai@ntu.edu.tw

* Corresponding author

To study the effect of slice number, we generate expression levels of gene sets along with a continuous phenotype as the third simulation study in the article. Gene expressions are generated according to the following model: For $i = 1, \ldots, n$,

$$X_i \stackrel{i.i.d.}{\sim} MVN(0, \Sigma_X),$$

where the elements of the covariance matrix $\Sigma_X = (\rho_{i,j})_{p \times p}$ are given by

$$\rho_{i,j} = \begin{cases} 1, & 1 \leq i = j \leq p, \\ \rho, & 1 \leq i \neq j \leq p_1, \\ \rho^{|i-j|}, & p_1 + 1 \leq i \neq j \leq 2p_1, \\ 0, & \text{otherwise.} \end{cases}$$

That is, all p genes have unit variance, and first $2p_1$ of them are pairwisely correlated. The first p_1 genes are equi-correlated pairwisely with correlation ρ. The correlation of the next p_1 genes decreases as the distance between the two genes increases. Specifically, $\rho = 0, 0.3, 0.6, 0.9$ are selected.

For the null scenario, the continuous phenotype Y, being independent of X, is randomly drawn from $N(0, 1)$. We consider two alternative scenarios. The first is a traditional normal linear regression model : For $i = 1, \ldots, n$, given x_i,

$$Y_i | x_i \sim N(x_i^T \beta, 1).$$

The second alternative model is a non-linear model: For $i = 1, \ldots, n$, given x_i,

$$Y_i | x_i \sim N(exp(x_i^T \beta), 1).$$

In which, the regression coefficient vector is $\beta = (\beta_1, \ldots, \beta_p)^T$. Suppose that in both models the phenotype Y depends on ten genes, five belong to the first group of p_1 genes, the other five belong to the next p_1 genes. We randomly select 5 out of the first p_1 genes, then produce their corresponding β_j's from $N(\nu, |\nu|)$. Next, another 5 genes out of the second p_1 genes is randomly selected, and their corresponding $\beta_{j'}$'s are generated from $N(-\nu, |\nu|)$. Aside from

the ten selected genes, all other genes have zero regression coefficients. Several ν's ranging from 0 to 2 in increments of 0.2 are considered.

For total sample size 30, we consider slice number $H = 2, 3, 4, 5$. Figures S1 and S2 show the empirical power using a nominal level of 0.05 for SDR_T and SDR_V, respectively. It is clear that SDR_T is sensitive to the choice of slice number, but SDR_V is not. For both statistics, using fewer slice number yields better performances.

Figure S1. The power curve of SDR_T of the experiment with a continuous phenotype for $n = 30$, slice number $H = 2, 3, 4, 5$ at significant level $\alpha = 0.05$

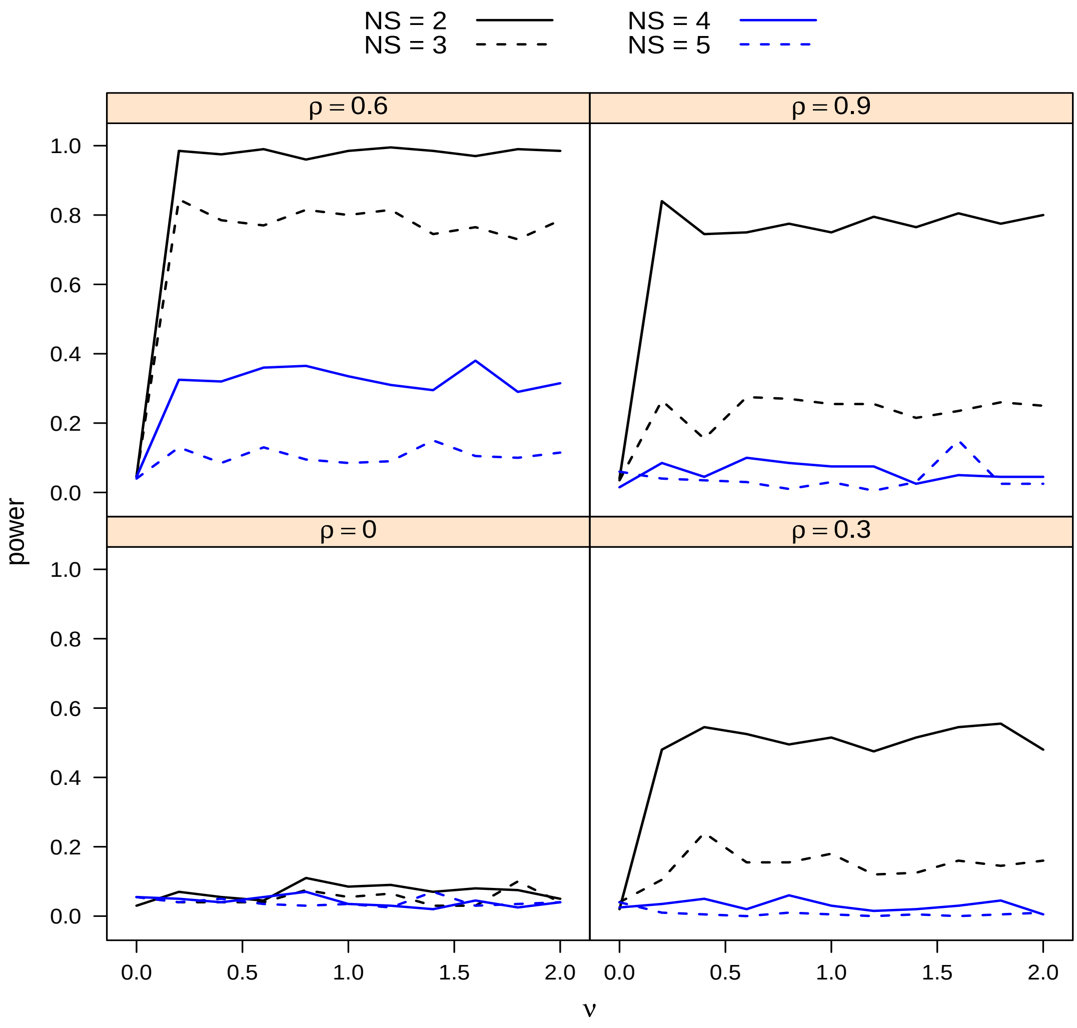

Figure S2: The power curve of SDR_V of the experiment with a continuous phenotype for $n = 30$, slice number $H = 2, 3, 4, 5$ at significant level $\alpha = 0.05$

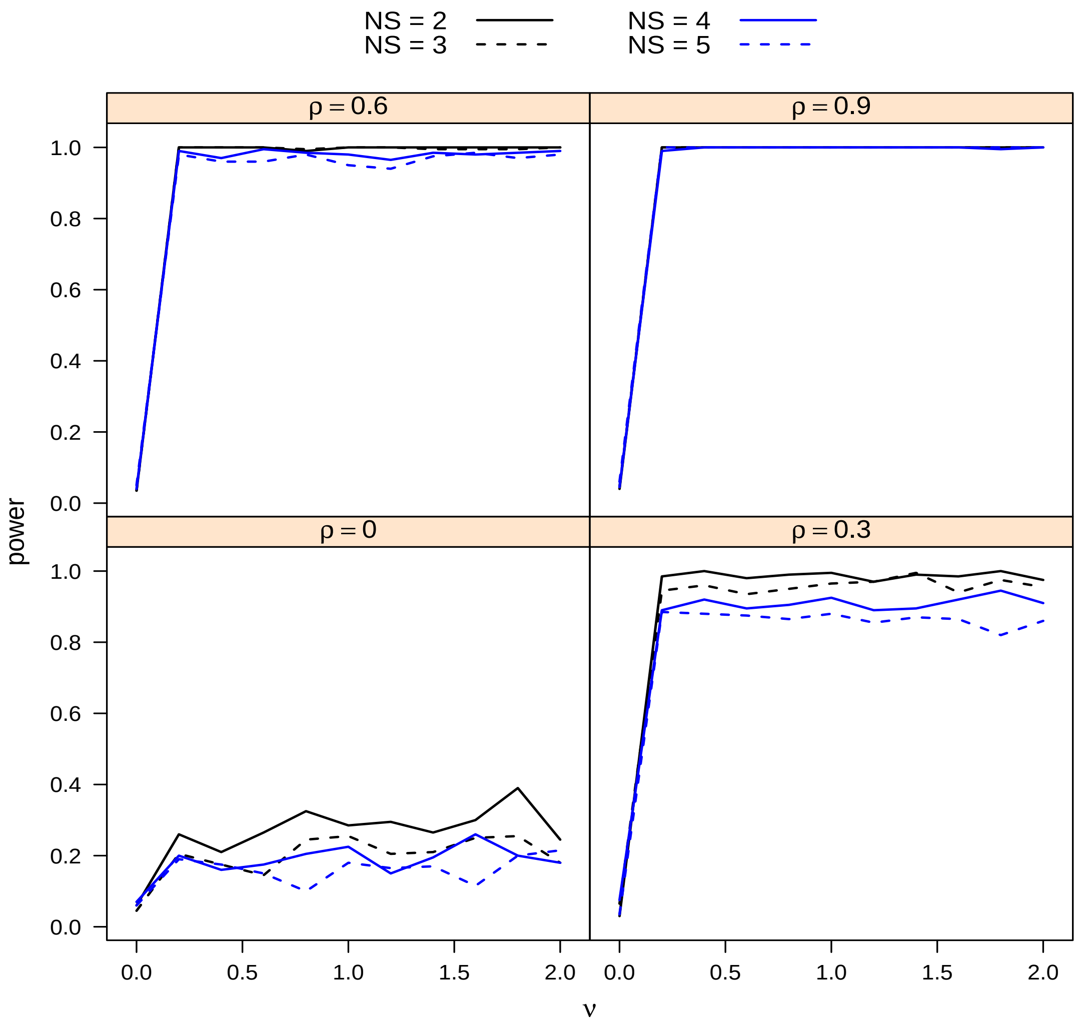

Supplement: Additional file 1 — The effect of slice numbers on SDR method. (PDF 126 kb) [file 12859_2016_928_MOESM1_ESM.pdf]
